# Effect of Repetitive Transcranial Magnetic Stimulation on Pain Management: A Systematic Narrative Review

**DOI:** 10.3389/fneur.2020.00114

**Published:** 2020-02-18

**Authors:** Seoyon Yang, Min Cheol Chang

**Affiliations:** ^1^Department of Rehabilitation Medicine, Ewha Woman's University Seoul Hospital, Ewha Woman's University School of Medicine, Seoul, South Korea; ^2^Department of Rehabilitation Medicine, College of Medicine, Yeungnam University, Daegu, South Korea

**Keywords:** repetitive transcranial magnetic stimulation, neuropathic pain, central pain, fibromyalgia, headache, musculoskeletal pain, complex regional pain syndrome

## Abstract

Recently, clinicians have been using repetitive transcranial magnetic stimulation (rTMS) for treating various pain conditions. This systematic narrative review aimed to examine the use and efficacy of rTMS for controlling various pain conditions. A PubMed search was conducted for articles that were published until June 7, 2019 and used rTMS for pain alleviation. The key search phrase for identifying potentially relevant articles was (repetitive transcranial magnetic stimulation AND pain). The following inclusion criteria were applied for article selection: (1) patients with pain, (2) rTMS was applied for pain management, and (3) follow-up evaluations were performed after rTMS stimulation to assess the reduction in pain. Review articles were excluded. Overall, 1,030 potentially relevant articles were identified. After reading the titles and abstracts and assessing eligibility based on the full-text articles, 106 publications were finally included in our analysis. Overall, our findings suggested that rTMS is beneficial for treating neuropathic pain of various origins, such as central pain, pain from peripheral nerve disorders, fibromyalgia, and migraine. Although data on the use of rTMS for orofacial pain, including trigeminal neuralgia, phantom pain, low back pain, myofascial pain syndrome, pelvic pain, and complex regional pain syndrome, were promising, there was insufficient evidence to determine the efficacy of rTMS for treating these conditions. Therefore, further studies are needed to validate the effects of rTMS on pain relief in these conditions. Overall, this review will help guide clinicians in making informed decisions regarding whether rTMS is an appropriate option for managing various pain conditions.

## Introduction

Transcranial magnetic stimulation (TMS) is a safe, non-invasive technique that uses an electromagnetic coil to generate a magnetic field. TMS can stimulate the brain cortex by producing brief magnetic pulses that pass easily and painlessly through the skull and into the brain. These pulses induce changes in cortical excitability at the stimulation site and transynaptically at distant areas (1, 2). Repeatedly applying TMS pulses is termed repetitive TMS (rTMS). rTMS has been proven effective in improving motor and cognitive functions and reducing depressive symptoms in several disorders, including stroke, Parkinson's disease, and major depressive disorder (3). Moreover, rTMS induces pain reducing effects in various pain conditions (4).

Pain is defined as unpleasant sensory and emotional experiences that are associated with actual or potential tissue damage (2). Although pain is most often related to a disease or injury, it may also be associated with one's emotional state. Several brain areas, including the hypothalamus, amygdala, thalamus, somatosensory cortex, insula, anterior cingulate cortex, and prefrontal cortex, are related with the experience of pain (2). Patients with painful conditions have various clinical outcomes. Acute pain conditions resolve over time after the normal healing process, but in some cases, they progress to chronic pain conditions that impact patients' quality of life (QoL) (5). Moreover, some patients with chronic pain do not respond to various conventional treatments, including drugs, injections with anesthetics and corticosteroids, and behavioral therapies (6). Recently, various neurostimulation methods, including rTMS, peripheral nerve stimulation, spinal cord stimulation, deep brain stimulation, and motor cortex stimulation, have been applied to chronic-pain treatments (7).

Among these methods, rTMS is a cortical stimulation technique that has been applied to modulate abnormal brain activities to alleviate pain. The mechanism of cortical stimulation for pain relief is based on the modification of neuronal excitability. rTMS is postulated to induce alterations in the activity of cortical and subcortical brain structures that are related to pain modulation and processing, including the orbitofrontal cortices, medial thalamus, anterior cingulate, and periaqueductal gray matter (8). Additionally, rTMS reduces chronic pain by triggering descending inhibitory neural pathways to act at the dorsal-horn level (9). Specifically, rTMS is known to alter neuronal activities in the periaqueductal gray matter, which is related to pain processing (10). Stimulation frequency is associated with synaptic changes; higher frequencies (> 5 Hz) are excitatory, and lower frequencies (< 1 Hz) are inhibitory. Thus, high-frequency stimulation increases cortical excitability, while low-frequency stimulation decreases cortical excitability (11). The stimulation frequency can be applied differently according to the stimulation site and patient pain conditions.

Herein, we review previous studies to investigate the effectiveness of rTMS for controlling various types of painful conditions.

## Methods

We searched the MEDLINDE database (PubMed) for articles that were published until June 7, 2019 that used rTMS to treat pain. The key search phrase for identifying potentially relevant articles was [repetitive transcranial magnetic stimulation AND pain]. The following inclusion criteria were applied for the selection of articles: (1) patients with pain, (2) rTMS was applied for controlling pain, and (3) follow-up evaluations were performed after rTMS stimulation to assess the degree of pain reduction. We excluded the following types of studies: (1) reviews, (2) animal studies, and (3) conference abstracts or presentations.

## Results

**Figure 1:**
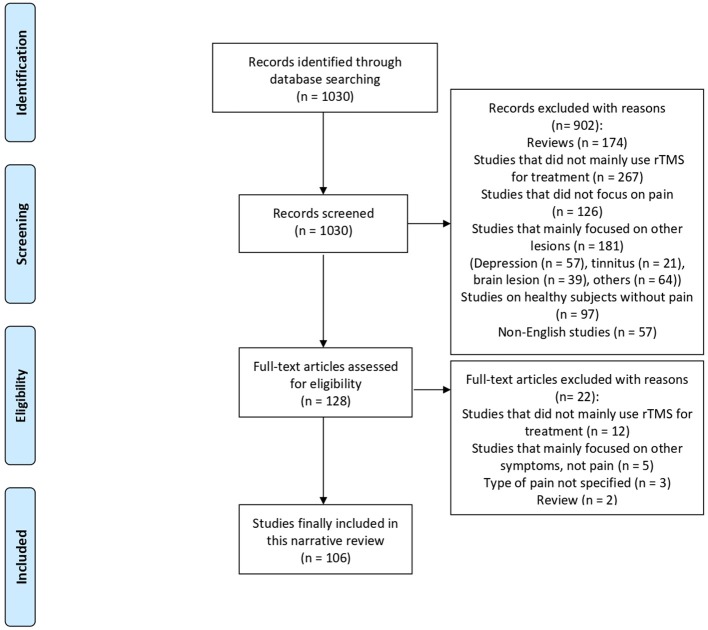
Flow chart showing the search results for the study.

We identified 1,030 potentially relevant articles. The titles and abstracts of the papers were screened to determine eligibility. Next, full-text articles were retrieved to verify study eligibility, and a total of 106 publications were finally included in this review (Figure 1). These publications consisted of non-randomized, observational, and randomized studies (parallel or crossover design); their characteristics are summarized in Table 1.

**Table 1:** rTMS protocols.

**#**	**References**	**Study design**	**Number of patients (E/C)**	**Frequency (Hz)**	**Intensity (MT, %)**	**Parameters and Dosage**	**Coil Type**	**Stimulation Site**	**Session schedule**	**Outcome measure**
**NEUROPATHIC PAIN**										
1	Lefaucheur et al. (12)	Cross-over	18 (18/18)	0.5 Hz/10Hz	80% RMT	50 pulse, 20 trains/session, total 1,000 pulses/session, ITI = 55 s	Figure 8	M1	3 rTMS sessions at 0.5, 10 Hz	VAS
2	Rollnik et al. (13)	Cross-over	12 (12/12)	20 Hz	80% RMT	40 pulses, 20 trains/session, total 800 pulses/session, ITI = 58 s	Figure 8	Contralateral M1	Single e rTMS session	VAS
3	Lefaucheur et al. (14)	Cross-over	60 (60/60)	10 Hz	80% RMT	20 trains/session, ITI = 55 s	Figure 8	M1	Single rTMS session	VAS
4	Andre-Obadia et al. (15)	Cross-over	12 (12 1/12 20 Hz/12 sham)	1/20 Hz	90% RMT	80 pulses, 20 trains/session, total 1,600 pulses/session 200, ITI = 84 s	Figure 8	M1	3 rTMS sessions at 1, 20 Hz	VAS
5	Hirayama et al. (16)	Cross-over	20 (20/20)	5 Hz	90% RMT	50 pulses, 10 trains/session, total 500 pulses/session, ITI = 50 s	Figure 8	M1, S1, PMC, SMA	4 rTMS sessions	VAS
6	Lefaucheur et al. (17)	OLT	36 (36/36)	10 Hz	90% RMT	20 trains/session, ITI = 50s	Figure 8	M1	2–3 rTMS sessions	VAS
7	Lefaucheur et al. (18)	Cross-over	46 (46/46)	1/10 Hz	90% RMT	Total 1,200 pulses/session, ITI = 54s	Figure 8	M1	3 rTMS sessions at 1, 10 Hz	VAS
8	Andre-Obadia et al. (19)	Cross-over	28 (28/28)	20 Hz	90% RMT	80 pulses × 20 trains, total 1,600 pulses/session, ITI = 84 s	Figure 8	M1 (posteroanterior vs. lateromedial)	2 rTMS sessions	VAS
9	Borckardt et al. (20)	Cross-over	4 (4/4)	10 Hz	100% RMT	Total 4,000 pulses/session, ITI = 20 s	Figure 8	Left PFC	3 rTMS sessions	NRS
10	Sampson et al. (21)	OLT	9	1 Hz	110% MT	Total 1,600 pulses/session	NA	Right DLPFC	15 rTMS sessions	VAS
11	Lefaucheur et al. (22)	OLT	14 (14/14)	10 Hz	90% AMT	100 pulses, 20 trains/session, total 2,000 pulses/session, ITI = 30 s	Figure 8	Contralateral M1	3 rTMS sessions	VAS
12	Hosomi et al. (23)	Cross-over	64 (64/64)	5 Hz	90% RMT	50 pulses × 10 trains/sessions, total 500 pulses/session, ITI = 50 s	Figure 8	M1	10 rTMS sessions	VAS
13	Onesti et al. (24)	Cross-over	23 (23/23)	20 Hz	100% RMT	50 pulses, 30 trains/session, total 1,500 pulses/session, ITI = 30 s	H-coil	M1	5 rTMS sessions	VAS
14	Khedr et al. (25)	Parallel	30 (15/15)	20 Hz	80% RMT	200 pulse, 10 trains/session, total 2,000 pulses/session, ITI = 30 s	Figure 8	M1	10 rTMS sessions	VAS
15	Attal et al. (26)	Parallel	35 (23/12)	10 Hz	80% RMT	100 pulses, 30 trains/session, total 3,000 pulses/session, ITI = 20 s	Figure 8	M1	3 rTMS sessions	NRS
16	Ayache et al. (27)	Cross-over	66	10 Hz	90% RMT	10 pulses, 30 trains/session, total 3,000 pulses/session, ITI = 20 s	Figure 8	M1	2 rTMS sessions	VAS
17	Nurmikko et al. (28)	Cross-over	27 (27/27)	10 Hz	90% RMT	20 pulses, 20 trains/session, total 2,000 pulses/session, ITI = 60 s	Figure 8	M1 (hotspot vs. alternative site)	5 rTMS sessions	NRS
18	Pommier et al. (29)	OLT	40	20 Hz	80% RMT	80 pulses, 20 trains/session, total 1,600 pulses/session, ITI = 84 s	Figure 8	Contralateral M1	Mean 11 sessions days (range 1–37 sessions)	VAS, percentage of pain relief
19	Shimizu et al. (30)	Cross-over	18 (18/18)	5 Hz	90% RMT	50 pulses, 10 trains/session, total 500 pulses/session, ITI = 50s	Figure 8 vs. H-coil	M1 to painful lower limb	5 rTMS sessions per group	VAS
20	Andre-Obadia et al. (31)	Cross-over	32 (32/32)	20 Hz	90% MT	80 pulses, 20 trains/session, total 1,600 pulses/session, ITI = 84 s	Figure 8	M1	2 rTMS sessions	NRS
21	Hodaj et al. (32)	Case report	1	10 Hz	80% RMT	50 pulses, 40 trains/session, total 2,000 pulses/session, ITI = 25 s	Figure 8	Vertex	12 rTMS sessions	NRS
22	Lawson et al. (33)	OLT	50	10 Hz	80% RMT	10 trains/session, ITI = 50s	Figure 8	Contralateral M1	9 rTMS sessions	VAS
**CENTRAL PAIN AFTER STROKE**										
23	Migita et al. (34)^*^	Case report	2	NR	NA	NA	NA	NA	NA	NA
24	Lefaucheur et al. (35)	Cross-over	14 (14/14)	10 Hz	80% RMT	50 pulses, 20 trains/session, total 1,000 pulses/session, ITI = 55 s	Figure 8	M1	Single rTMS session	VAS
25	Khedr et al. (36)	Parallel	24 (14/10)	20 Hz	80% RMT	200 pulses, 10 train/session, total 2,000 pulses/session	Figure 8	M1	5 rTMS sessions	VAS
26	Saitoh et al. (37)	Cross-over	13 (13/13)	1/5/10 Hz	90% RMT	Total 5,000 pulses/session, ITI = 50 s	Figure 8	M1	3 rTMS sessions	VAS
27	Ohn et al. (38)	OLT	14 (14/14)	10 Hz	90% RMT	50 pulses, 20 trains/session, total 1,000 trains/session, ITI = 55 s	Figure 8	M1	5 rTMS sessions	VAS
28	Matsumura et al. (39)	Cross-over	20 (20/20)	5 Hz	100% RMT	50 pulses, 10 trains/session, total 500 pulses/session, ITI = 25 s	Figure 8	M1	Single rTMS session	VAS
29	Hasan et al. (40)	OLT	14	10 Hz	80–90% MT	Total 2,000 pulses/session, ITI = 60 s	Figure 8	M1	5 rTMS sessions	NRS
30	de Oliveira et al. (41)	Parallel	21 (11/10)	10 Hz	120% RMT	50 pulses, 25 trains/session, total 1,250 pulses, ITI = 25	Figure 8	Left PMC/DLPMC	10 rTMS sessions	VAS
31	Kobayashi et al. (42)	OLT	18	5 Hz	90% AMT	50 pulses, 10 trains/session, total 500 pulses, ITI = 50 s	Figure 8	M1	12 rTMS sessions	VAS
32	Choi et al. (4)	Parallel	12 (6/6)	10 Hz	90% MT	50 pulses, 20 trains/session, total 1,000 pulses, ITI = 55 s	Figure 8	M1	10 rTMS sessions	NRS
33	Lin et al. (43)	OLT	7	10 Hz	90% RMT	100 pulses, 10 trains/session, total 1,000 pulses, ITI = 60 s	Figure 8	M1	10 rTMS sessions	VAS
**CENTRAL PAIN AFTER SPINAL CORD INJURY (SCI)**										
34	Defrin et al. (44)	Parallel	11 (6/5)	5 Hz	115% MT	500 pulses, 1 train/session, total 500 pulses/session, ITI = 30 s	Figure 8	M1	10 rTMS sessions	VAS
35	Kang et al. (45)	Cross-over	11 (11/11)	10 Hz	80% RMT	50 pulses, 20 trains/session, total 1,000 pulses/session, ITI = 55 s	Figure 8	M1	5 rTMS sessions	NRS
36	Jette et al. (46)	Cross-over	16 (16/16)	10 Hz	90% RMT/110% RMT	50 pulses, 40 trains/session, total 2,000 pulses/session, ITI = 25 s	Figure 8	M1 (hand vs. leg area)	Single rTMS session	NRS
37	Yilmaz et al. (47)	Parallel	17 (9/8)	10 Hz	110% RMT	50 pulses, 30 trains, total 1,500 pulses/session, ITI = 25 s	Figure 8	Vertex	10 rTMS sessions	VAS
38	Nardone et al. (48)	Parallel	12 (6/6)	10 Hz	120% RMT	50 pulses, 25 trains, total 1,250 pulses, ITI = 25 s	Figure 8	Left PMC/DLPFC	10 rTMS sessions	VAS
**CENTRAL PAIN AFTER STROKE OR SCI**										
39	Quesada et al. (49)	OLT	71	20 Hz	80% MT	80 pulses × 20 trains/sessions, total 1,600 pulses/session, ITI = 84 s	Figure 8	Contralateral M1	4 rTMS sessions	NRS
40	Galhardoni et al. (50)	Parallel	98 (33 ACC /33 PSI/32 sham)	10 Hz	120% RMT (FDI)	100 pulses × 15 trains/sessions, total 1,500 pulses/session, ITI = 50 s	Figure 8	ACC vs. PSI	16 rTMS sessions	NRS
**FIBROMYALGIA**										
41	Sampson et al. (51)	OLT	4	1 Hz	110% MT	Total 1,600 pulses/session, ITI = 60 s	Figure 8	Right DLPFC	18–20 rTMS sessions	NRS
42	Passard et al. (52)	Parallel	30 (15/15)	10 Hz	80% RMT	Total 2000 pulses/session, ITI = 52 s	Figure 8	Left M1	10 rTMS sessions	NRS
43	Carretero et al. (53)	Parallel	26 (14/12)	1 Hz	110% MT	Total 1,200 pulses/session, ITI = 45 s	Figure 8	Right DLPFC	20 rTMS sessions	Likert pain scale
44	Mhalla et al. (54)	Parallel	30 (16/14)	10 Hz	80% RMT	Total 1500 pulses/session, ITI = 50s	Figure 8	Left M1	15 rTMS sessions	NRS
45	Short et al. (55)	Parallel	20 (10/10)	10 Hz	120% RMT	Total 4000 pulses/session, ITI = 10 s	Solid focal coil	Left DLPFC	10 rTMS sessions	NRS
46	Lee et al. (56)	Parallel	15 (5 1 Hz/5 10 Hz/5 sham)	1/10 Hz	110% RMT/80% RMT	Total 1600 pulses/session, ITI = 60 s/total 2000 pulses/session, ITI = 10 s	Figure 8	Right DLPFC/left M1	10 rTMS sessions	VAS
47	Maestu et al. (57)	Parallel	54 (28/26)	8 Hz	NR	NR	NR	NR	8 rTMS sessions	VAS
48	Tzabazis et al. (58)	Cross-over	16	1 Hz/10 Hz	110% MT	Total 1,800 pulses/session	Multi-coil	Configuration B and E as described in the study	20 rTMS sessions	NRS
49	Avery et al. (59)	Parallel	18 (7/11)	10 Hz	120% MT	40 pulses, 75 trains/session, total 3,000 pulses/session, ITI = 26s	NA	Left DLPFC	15 rTMS sessions	NRS, VAS
50	Altas et al. (60)	Parallel	30 (10 M1/10 DLPFC/10 sham)	10 Hz	90% RMT	Total 1,200 pulses/session	NA	Left M1 vs. DLPFC	15 rTMS sessions	VAS
51	Cheng et al. (61)	Parallel	20 (9/11)	10 Hz	100% MT	Total 1,600 pulses/session, ITI = 26 s	Figure 8	Left DLPFC	10 rTMS sessions	VAS
52	Abd Elghany et al. (62)	Parallel	120 (60/60)	10 Hz	NA	5 s duration, ITI = 10s	NA	Left DLPFC	15 rTMS sessions	VAS
**MIGRAINE**										
53	Brighina et al. (63)	Parallel	11 (6/5)	20 Hz	90% MT	10 trains/session, 2s duration, ITI = 30 s	Figure 8	Left DLPFC	12 rTMS sessions	Headache index
54	Clarke et al. (64)	OLT	42	High vs. low	NA	NA	NA	NA	3 sTMS sessions	Likert-type scale
55	O'Reardon et al. (65)^*^	Case reports	2	NA	NA	NA	NA	NA	2 rTMS sessions	-
56	Lipton et al. (66)	Parallel	164 (82/82)	sTMS	NA	Two pulses 30 s apart, rise time 180μs, total pulse length < 1 ms	NA	Occiput	sTMS	Global assessment of pain
57	Teepker et al. (67)	Parallel	27 (14/13)	1 Hz	Visual MT-2%	500 pulses, 2 trains/session, total 1,000 pulses, ITI = 60 s	Circular coil	Vertex	5 rTMS sessions	NRS
58	Misra et al. (68)	Parallel	158 (48/47)	10 Hz	70% MT	60 pulses, 10 trains/session, total 600 pulses, ITI = 45 s	Figure 8	Left frontal cortex	3 rTMS sessions	VAS
59	Misra et al. (69)	Parallel	45 (25/20)	10 Hz	70% MT	60 pulses, 10 trains/session, total 600 pulses, ITI = 45 s	Figure 8	M1	3 rTMS sessions	VAS
60	Conforto et al. (70)	Parallel	18 (9/9)	10 Hz	110% RMT	50 pulses, 32 trains/session, 5s duration, total 1,600 pulses/session, ITI = 30 s	Figure 8	Left DLPFC	23 rTMS sessions	MIDAS
61	Bhola et al. (71)	OLT	426	sTMS	NA	Rise time of 180μs, total pulse length < 1 ms	NA	Occiput	sTMS	NRS
62	Hodaj et al. (72)	OLT	55	10 Hz	80% MT	50 pulses, 40 trains/session, total 2,000 pulses/session, ITI = 25 s	Figure 8	Contralateral M1	12 rTMS sessions	VAS
63	Leung et al. (73)	Case report	6	10 Hz	80% MT	100 pulses, 20 trains/session, total 2,000 pulses/session	Figure 8	Left M1, DLPFC	4 rTMS sessions	NRS
64	Leung et al. (74)	Parallel	24 (12/12)	10 Hz	80% RMT	100 pulses, 20 trains/session, total 2,000 pulses/session, ITI = 1 s	Figure 8	Left M1	3 rTMS sessions	NRS
65	Rapinesi et al. (75)	Parallel	14 (7/7)	10 Hz	100% MT	36 pulses, 10 trains/session, total 360 pulses/session, ITI = 20 s	H1 coil	Left DLPFC	12 rTMS sessions	VAS
66	Shehata et al. (76)	Parallel	29 (14/15)	10 Hz	80% MT	100 pulses, 20 trains/session, total 2,000 pulses/session, ITI = 10 s	Figure 8	Left M1	12 rTMS sessions	VAS
67	Zardouz et al. (77)	Case report	5	10 Hz	80% RMT	100 pulses, 20 trains/session, total 2,000 pulses/session, ITI = 1 s	Figure 8	Left M1	5 rTMS sessions	NRS
68	Misra et al. (78)	Parallel	93 (24 single session/22 3 sessions/47 sham)	10 Hz	70% MT	60 pulses, 10 trains/session, total 600 pulses, ITI = 45 s	Figure 8	Left M1	1 vs 3 rTMS sessions	VAS
69	Leung et al. (79)	Parallel	29 (14/15)	10 Hz	80% RMT	100 pulses, 20 trains/session, total 2,000 pulses/session, ITI = 1 s	Figure 8	Left DLPFC	4 rTMS sessions	NRS
70	Sahu et al. (80)	Cross-over	41 (20/21)	5 Hz	80% AMT	30 pulses, 20 trains/session, total 600 pulses/session, ITI = 8 s	Figure 8	Left DLPFC	10 rTMS sessions	MIDAS
**OROFACIAL PAIN**										
71	Reid et al. (81)	Case report	1	20 Hz	100% MT	30 trains/session, 2 s duration	NA	Left PFC	14 rTMS sessions	VAS
72	Khedr et al. (36)	Parallel	24 (14/10)	20 Hz	80% RMT	200 pulses, 1 train/session, total 2,000 pulses/session	Figure 8	M1	5 rTMS sessions	VAS
73	Zaghi et al. (82)	Case report	1	10 Hz	NA	40 pulses, 30 trains/session, total 1,200 pulses/session, ITI = 26 s	NA	M1	35 rTMS sessions	NRS
74	Fricova et al. (83)	Parallel	23 (13/10)	20 Hz	95% MT	Total 720 pulses/session, ITI = 1.9 s	Figure 8	Contralateral M1	5 rTMS sessions	VAS
75	Lindholm et al. (84)	Cross-over	16 (16/16)	10 Hz	90% RMT	Total 1000 pulses/session, ITI = 10 s	Figure 8	Contralateral S1/M1 vs right S2	3 rTMS sessions at S1/M1, S2	NRS
76	Umezaki et al. (85)	Case report	1	10 Hz	110% RMT	Total 3,000 pulses/session, ITI = 10 s	Figure 8	Left DLPFC	10 rTMS sessions	VAS
77	Umezaki et al. (86)	Paralel	20 (12/8)	10 Hz	110% RMT	Total 3,000 pulses/session, ITI = 10 s	Figure 8	Left DLPFC	10 rTMS sessions	VAS
78	Henssen et al. (87)	Cross-over	12 (12/12)	10 Hz	80% RMT	10 pulses, 10 trains/sessions, total 1,000 pulses/session, ITI = 50 s	Figure 8	M1 (unilateral vs bilateral)	Single rTMS session each	VAS
**PHANTOM PAIN**										
79	Topper et al. (88)	Case report	2	1 Hz/10 Hz	110% RMT	1 train/session, 12 min duration (1 Hz) & 20 trains/session, 2 s duration, ITI = 60 s (10 Hz)	Figure 8	Contralateral parietal cortex	15 rTMS sessions	VAS
80	Ahmed et al. (89)	Parallel	27 (17/10)	20 Hz	80% RMT	200 pulses, 10 train/session, total 2,000 pulses/session, ITI = 50 s	Figure 8	M1	5 rTMS sessions	VAS
81	Di Rollo et al. (90)	Case report	1	1 Hz	80% RMT	20 pulses, 30 trains/session, total 600 pulses/session, ITI = 10 s	Figure 8	Left M1	15 rTMS sessions	VAS
82	Grammer et al. (91)	Case report, crossover	1	1 Hz/10 Hz	100% MT/120% MT	Total of 2000 pulses, ITI = 4s (1Hz) & total of 3,000 pulses, ITI = 26 s (10 Hz)	NA	Left PSC (1 Hz) /left DLPFC (10 Hz)	28 rTMS sessions days (17 LF, 11 HF stimulations)	VAS
82	Lee et al. (92)	Case report	1	1 Hz	85% RMT	Total 800 pulses/session	Figure 8	M1/SMC	10 rTMS sessions, 6 rounds of treatment	VAS
83	Malavera et al. (93)	Parallel	54 (27/27)	10 Hz	90% RMT	60 pulses × 20 trains/sessions, total 1,200 pulses, ITI = 54 s	Figure 8	Contralateral M1	10 rTMS sessions	VAS
84	Scibilia et al. (94)	Case report, crossover	1	1 Hz/10 Hz	NA	NA	NA	Left PSC (1 Hz) /DLPFC (10 Hz)	30 rTMS sessions	VAS
**LOW BACK PAIN**										
85	Park et al. (95)	Case report	2	1 Hz	100% RMT	Total 1,200 pulses/session	Figure 8	Left DLPFC	20 & 15 rTMS sessions	NRS
86	Ambriz-Tututi et al. (96)	Cross-over	82 (44/38)	20 Hz	95% RMT	10 pulse trains, 10 s duration, ITI = 28 s	Figure 8	M1	5 rTMS sessions	VAS
87	Yates et al. (97)	Case report	2	18 Hz	NA	2 s period of 18 Hz pulses followed by 20 s of down time	NA	NA	26 dTMS sessions	VAS
**MYOFASCIAL PAIN SYNDROME**										
88	Dall'Agnol et al. (98)	Parallel	24 (12/12)	10 Hz	80% RMT	100 pulses, 16 trains/session, total 1,600 pulses/session, ITI = 26 s	Figure 8	Left M1	10 rTMS sessions	VAS
89	Medeiros et al. (99)	Parallel	44 (11 rTMS/11 DIMST/11 rTMS+DIMST/11 sham)	10 Hz	80% RMT	Total 1,600 pulses/session	Figure 8	Left M1	10 rTMS sessions	VAS
**PELVIC PAIN**										
90	Cervigni et al. (100)	Cross-over	13 (13/13)	20 Hz	110% RMT	50 pulses, 30 trains/session, total 1,500 pulses/session, ITI = 30 s	H-coil	M1	10 rTMS sessions	VAS
91	Nizard et al. (101)	Case report	1	1 Hz	110% RMT	Total 1,200 pulses/session	NA	Right then left DLPFC	16 rTMS sessions	NRS
92	Pinot-Monange et al. (102)	OLT	12	10 Hz	80% RMT	Total 1,500 pulses/session, ITI = 50 s	Figure 8	Left M1	5 rTMS sessions	VAS
**COMPLEX REGIONAL PAIN SYNDROME**										
93	Pleger et al. (103)	Cross-over	10 (10/10)	10 Hz	110% RMT	10 trains/session, 1.2 s duration, ITI = 10 s	Figure 8	Contralateral M1	Single rTMS session	VAS
94	Picarelli et al. (104)	Parallel	22 (11/11)	10 Hz	100% RMT	100 pulses, 25 trains/session, total 2,500 pulses/session, ITI = 60 s	Figure 8	M1	10 rTMS sessions	VAS
95	Gaertner et al. (105)	OLT	17 (5/12)	10 Hz	80% RMT	Total 2,000 pulses/session, ITI = 30 s	Figure 8	M1	1 vs 5 rTMS sessions	VAS, NRS
**OTHERS**										
96	Lefaucheur et al. (14) BPI	Case report	1	10 Hz	80% RMT	20 trains/session, 5 s duration, ITI = 55 s	Figure 8	M1	16 rTMS sessions	VAS
97	Borckardt et al. (106) post-surgical pain	Parallel	20 (10/10)	10 Hz	100% RMT	Total 4,000 pulses/session, ITI = 20 s	Figure 8	Left PFC	Single rTMS session	VAS, morphine use
98	Borckardt et al. (107) post-surgical pain	Parallel	20 (10/10)	10 Hz	100% RMT	Total 4,000 pulses/session, ITI = 20 s	Figure 8	Left PFC	Single rTMS session	VAS, morphine use
99	Fregni et al. visceral pain (108)	Parallel	17 (9/8)	1 Hz	NA	Total 1,600 pulses/session	Figure 8	S2	10 rTMS sessions	VAS
100	Bertolucci et al. (109) BPI	Case report	1	10 Hz	80% RMT	Total 800 pulses/session, ITI = 52 s	Figure 8	M1	10 rTMS sessions	VAS
101	Borckardt et al. (110) post-surgical pain	Parallel	108 (28 rTMS, 52 rTMS+sham, 28 sham)	10 Hz	100% RMT	Total 4,000 pulses/session, ITI = 20 s	Figure 8	Left DLPFC	2 rTMS sessions, 1 rTMS + 1 sham	VAS, morphine use
102	Qiu et al. (111) BPI	Case report	1	20 Hz	120% RMT	Total 2,000 pulses/session	Circular coil	Contralateral M1	20 rTMS sessions	VAS
103	Ma et al. (112) PHN	Parallel	40 (20/20)	10 Hz	80% RMT	Total 1,500 pulses/session, ITI = 3 s	Figure 8	M1	10 rTMS sessions	VAS
104	Choi et al. (11) hemiplegic shoulder	Parallel	24 (12/12)	10 Hz	90% MT	Total 1,000 pulses/session, ITI = 55 s	Figure 8	M1	10 rTMS sessions	NRS
105	Singh et al. (113) SPD	Case report	5	10 Hz	100% MT	40 pulses, 30 trains/session, total 1,200 pulses/session, ITI = 26 s	Figure 8	Left DLPFC	18 rTMS sessions	VAS
106	Nguyen et al. (114) knee OA	Case report	1	10 Hz	80% RMT	70 pulses, 20 trains/session, total 1,400 pulses/session, ITI = 55 s	Figure 8	Contralateral M1	10 rTMS sessions	NRS

*ACC, anterior cingulate cortex; AMT, active motor threshold; C, control group; DIMST, deep intramuscular stimulation therapy; DLPFC, dorsolateral prefrontal cortex; dTMS, deep transcranial magnetic stimulation; E, experimental group; Figure 8, figure-of-8 coil; ITI, inter-train interval; M1, motor cortex; MIDAS, Migraine Disability Assessment Scale; MT, motor threshold; NA, not available; NR, not reported; NRS, numerical rating scales; OLT, open-label trial; PMC, premotor cortex; PSI, posterior superior insula; PSC, primary sensory cortex; PFC, prefrontal cortex; RMT, resting motor threshold; rTMS, repetitive transcranial magnetic stimulation; s, second; S1, postcentral gyrus; S2, secondary somatosensory cortex; SMA, supplementary motor area; SMC, supplementary motor complex; sTMS, single-pulse transcranial magnetic stimulation; VAS, visual analog scale; BPI, brachial plexus injury; PHN, postherpetic neuralgia; SPD, somatoform pain disorders; OA, osteoarthritis*.

* *Only abstract was available, obtainable data limited*.

## Discussion

### Neuropathic Pain

#### Neuropathic Pain of Various Origins

Neuropathic pain (NP) is a localized sensation of unpleasant discomfort that originates from a lesion or disease of the peripheral or central somatosensory system (115). NP symptoms include abnormal sensations (dysesthesia) and pain from non-painful stimuli (allodynia). Pharmacotherapy is often initially applied to patients with NP, but in many cases, drugs are ineffective and high doses are required for pain relief (115). NP is characterized by neuronal overexcitability and diverse features under various medical conditions. Aberrant regeneration after nerve lesions leads to abnormal neuronal excitability, and this increases sensitivity to various stimuli (116). Recently, it has been suggested that rTMS may be a safe and alternative treatment method that relieves NP via modulating cortical excitability and the pain threshold.

Our literature search located 19 studies that examined the clinical usefulness of rTMS to control chronic NP (12–23, 27–33). Among the 19 studies, two were randomized controlled trials (RCTs) (19, 23). In 2008, Andre-Obadia et al. conducted a crossover RCT (19) that included 28 patients with NP in order to compare differences in pain relief between patients who received high-frequency rTMS and patients who received sham stimulation and to assess the effect of different coil orientations (posteroanterior vs. lateromedial) on pain relief The results show that 14 of the 28 patients who received posteroanterior rTMS of the motor cortex (M1) reported “satisfactory” global pain scores that lasted for ~1 week. Conversely, only seven of the 28 patients reported “satisfactory” global pain scores after sham stimulation. Additionally, the coil orientation change from posteroanterior to lateromedial did not result in significant pain relief. The 2013 crossover RCT by Hosomi et al. (23) recruited 70 patients from seven centers in Japan, and each patient was assigned to one of two treatment groups. A real rTMS period was followed by a sham period in one group, and a sham period was followed by a real rTMS period in the other group. After completing 10 stimulation sessions, follow-up evaluations of at least 17 days were recorded. In this study, patients' scores on a visual analog scale (VAS) and the short form of the McGill pain questionnaire showed significant short-term improvements during the 5-Hz rTMS sessions over the M1 as compared with those during the sham sessions.

The other studies included in our analysis were 16 prospective observational studies (12–18, 20–22, 27–31, 33) and one case study (32). Lefaucheur et al. conducted five studies that included patients with NP from 2001 to 2012 (12, 14, 17, 18, 22). Results from these studies showed that 10-Hz rTMS sessions effectively reduced pain according to the VAS. A study conducted by Hirayama et al. (16) compared the effectiveness of different rTMS targets with 20 patients, including the M1, post-central gyrus, premotor area, and supplementary motor area. They reported that the M1 was the sole effective target for treating intractable pain. In 2011, Sampson et al. (21) conducted low-frequency rTMS (1 Hz, 1,600 pulses/session) over the right dorsolateral prefrontal cortex (DLPFC) in nine patients with NP and showed that low-frequency rTMS was also effective in rapid-onset pain relief. In 2016, three more studies (27–29) supported that rTMS stimulation over the M1 effectively relieved NP. Furthermore, studies conducted in 2017 and 2018 reported that high-frequency (5–20 Hz) stimulations effectively controlled NP (30, 31, 33). In contrast to these positive results, two prospective observational studies (13, 15) and one case study (32) reported that there were no differences between rTMS and sham stimulations on NP relief.

The limitation of these studies is that they were conducted with populations of patients with different types of NP and various etiologies. Although they all defined NP according to the International Association for the Study of Pain criteria, the heterogeneity of the pool should be considered when summarizing the overall efficacy of rTMS for NP.

To provide more reliable and reproducible data, some RCTs were exclusively conducted on NP with specific etiologies. These studies included patients with NP due to a single disease. In 2013, Onesti et al. (24) conducted a crossover RCT that included 25 patients with NP due to diabetic symmetric polyneuropathy. Patients received five sessions of either 20-Hz rTMS (total 1,500 pulses/session) or sham rTMS. After a 5-week washout period, they crossed over to the alternative treatment for five additional sessions. After real rTMS, the VAS scores decreased significantly (*p* = 0.01), whereas there were no effects on VAS scores following sham rTMS. Additionally, this effect lasted for ~3 weeks after stimulation. In a study conducted by Khedr et al. (25), 30 patients with cancer and malignant NP received either real or sham rTMS (15 patients in each group). The effects of 10 rTMS sessions over the M1 (20 Hz, 2,000 pulses/session) on pain relief were greater than the effects of the sham sessions on pain relief. Further, this effect persisted for up to 15 days; however, it was not present 1 month later. A 2016 RCT by Attal et al. (26) exclusively included 35 patients who had NP due to radiculopathy. Twenty-three patients in the active treatment group received rTMS followed by transcranial direct-current stimulation (tDCS) or tDCS followed by active rTMS (crossover design), and 12 patients received sham stimulation. Results from this study demonstrated by pain intensity [measured by a numerical rating scale (NRS) and cold pain thresholds] were decreased after rTMS as compared with those after tDCS and sham stimulation.

Although some previous studies did not show positive pain reducing effects after rTMS, many others have shown the positive therapeutic effects of rTMS on NP. This suggests that rTMS may be a beneficial method for alleviating NP.

#### Central Pain

Central pain, which is characterized by NP, is associated with a burning sensation and hyperpathia and occurs in 10–30% of patients with brain and spinal injuries (4, 49). Patients with central pain also complain of various symptoms, including tingling, numbness, chilling, itching, and abnormal sensations. Central pain is caused by a lesion or dysfunction of the somatosensory pathways in the central nervous system (CNS), which is most commonly caused by stroke or spinal cord injury (SCI).

#### Central Pain After Stroke

Central poststroke pain (CPSP) is characterized by constant or intermittent pain that occurs after stroke and is associated with sensory abnormalities, including hypoesthesia, hyperesthesia, allodynia, and hyperalgesia (117). It is a chronic NP syndrome caused by lesions of the spinothalamocortical pathways. Patients often complain of spontaneous and evoked pain that is associated with the disruption of somatic sensations. CPSP occurs in 2–8% of stroke survivors and is present in up to 50% of patients with lesions that solely affect the spinothalamic pathways (118). In clinical practice, many cases of CPSP are refractory to medical treatment and difficult to manage. Thus, attempts to relieve pain with rTMS have been made, but the efficacy of rTMS in CPSP remains unclear (42).

Eleven studies, including three RCTs (4, 36, 41), seven prospective observational studies (35, 37–40, 42, 43), and one case study (34), evaluated the efficacy of rTMS in patients with central pain after cerebral lesion. In one of the RCTs, Khedr et al. (36) showed that CPSP was relieved in 24 patients who received five daily sessions of 20-Hz rTMS over the hand area of the M1 as compared with that in 10 patients who received sham stimulation. This effect was maintained 2 weeks after the end of treatment. Conversely, the RCT conducted by de Oliveira et al. (41), which compared the analgesic effect of left M1 and DLPFC rTMS with sham stimulation, showed that CSPS was not improved in patients who received 10 daily sessions of 10-Hz rTMS (11 patients) as compared with CSPS in patients who received sham (10 patients) stimulation. Interestingly, Choi et al. conducted an RCT in 2018 (4) and recruited 12 patients with chronic central pain after mild traumatic brain injury (six patients to either an rTMS or sham group). They showed that pain and QoL were improved after 10 sessions of 10-Hz rTMS over the M1 as compared with pain and QoL after sham stimulation. In that study, the NRS was used to evaluate pain intensity and the Short Form 36 Health Survey (SF-36), including the physical component score and the mental component score, was used to assess physical and mental health status, respectively. The NRS scores were significantly decreased and the SF-36 scores were significantly increased in the rTMS group after 10 rTMS sessions and at 1, 2, and 4 weeks after the rTMS sessions as compared with the NRS scores and SF-36 scores in the sham group.

Seven prospective observational studies (35, 37–40, 42, 43) also showed positive results of rTMS application in CPSP. Hasan et al. (40) reported that after five 10-Hz rTMS sessions, the NRS scores decreased from 7 to 6, and the improvement in the NRS scores were maintained for up to 4 weeks in 14 patients with CPSP who were involved in this study. Similarly, in 2015, Kobayashi et al. (42) reported that the application of 5-Hz rTMS over the M1 on the affected side of 18 patients with CPSP once a week effectively reduced pain for up to 8 weeks, and it remained effective in 61.1% of patients for up to 12 weeks. Most recently, Lin et al. (43) reported that VAS scores were significantly decreased after 10-Hz rTMS sessions over the M1 for 10 days in seven patients with thalamic pain. The VAS score decreased from 7 to 5.6 at 2 weeks and then to 3.9 at 8 weeks after rTMS treatment. Other observational studies that predominantly involved patients with CPSP (35, 37–39) also showed the analgesic effect of high-frequency rTMS stimulation (5–20 Hz).

#### Central Pain After SCI

Chronic pain after SCI affects more than 80% patients in the 5 years after trauma and results in chronic NP in up to 59% of individuals (119). The treatment of NP in patients with SCI is challenging because pharmacological and interventional therapies often show limited success. NP is considered the most resistant type of pain to treat in patients with SCI (120).

Three RCTs (44, 47, 48) and two prospective observational studies (45, 46) revealed that rTMS effectively managed chronic central pain in patients with SCI. Among the RCTs, Nardone et al. (48) performed 10 sessions of 10-Hz rTMS stimulation over the left prefrontal cortex in 12 patients with complete or incomplete motor SCI. The VAS scores of six patients in the real rTMS group decreased over time, and the differences in VAS scores between patients in the real rTMS group and the six patients in the sham group showed that rTMS effectively relieved pain in patients with SCI after 2 weeks of treatment. A prospective observational study conducted by Jette et al. (46) that measured pain with the NRS also revealed that rTMS sessions to either the hand or leg area induced pain reduction for 48 h in 16 patients with motor SCI with chronic NP.

Conversely, an RCT conducted by Defrin et al. (44) showed that both real rTMS (5 Hz, 500 pulses/session, 10 sessions, six patients) and sham rTMS (five patients) induced significant decreases in the VAS scores of 12 patients with thoracic SCI and chronic central pain. This study suggested that pain alleviation after rTMS may be due to placebo. Similarly, an RCT by Yilmaz et al. (47) concluded that 10 sessions of real 10-Hz rTMS (nine patients) and sham rTMS (eight patients) provided an equally significant reduction in the VAS scores, and the effect of rTMS was not superior to that of placebo on intractable NP in patients with SCI. One prospective observational study by Kang et al. (45) also reported that there was no difference in the changes in the NRS scores between real and sham rTMS in 11 patients with SCI and chronic central pain.

Recently, two additional studies have examined the efficacy of rTMS in patients with chronic NP after stroke or SCI. These studies enrolled patients with either stroke or SCI and did not distinguish between these two different disease entities. In 2019, Galhardoni et al. (50) conducted an RCT that included 98 patients with chronic NP that occurred after stroke or SCI to compare the differences in the analgesic effects between patients who received deep stimulation of the anterior cingulated cortex (ACC) or the posterior superior insula (PSI) and patients who received sham stimulation. Patients were allocated to the ACC-rTMS (33 patients), PSI-rTMS (33 patients), and sham rTMS (32 patients) groups. Although there were no differences in pain relief between patients who received rTMS and patients who received sham stimulation, the researchers observed that the antinociceptive effects after PSI stimulation and the anxiolytic effects after ACC were increased as compared with those after sham stimulation. Conversely, a prospective observational study conducted by Quesada et al. (49) reported that four sessions of 20-Hz rTMS over the M1 effectively alleviated chronic NP, and the effect was maintained over 12 months in 71 patients who were diagnosed with brain or SCI.

The results of these studies suggest that rTMS may have beneficial effects for controlling CPSP. However, the evidence for the utility and efficacy of rTMS for central pain after SCI appears insufficient. Therefore, additional prospective clinical studies should be conducted to clarify the clinical effects of rTMS in patients with central pain.

#### Fibromyalgia

Fibromyalgia (FM) is a chronic, widespread pain disorder that can develop at any age. FM is usually accompanied by multiple symptoms, including stiffness, fatigue, sleep disturbance, cognitive dysfunction, and depression. The modified version of the ACR diagnostic criteria was developed in 2010 for a more accurate diagnosis of this disease (121). Although rTMS has been used to treat chronic widespread pain since the 2000s, the beneficial effects of rTMS on FM remain controversial.

Our literature search revealed nine RCTs (52–57, 59–61) and three prospective observational studies (51, 58, 62) that investigated the usefulness of rTMS for treating patients with FM. Among them, six RCTs (52, 54, 56, 57, 60, 61) concluded that rTMS effectively reduced pain in patients with FM. Three RCTs (52, 54, 56) compared differences in pain and QoL between patients who received high-frequency rTMS stimulation (10 Hz) at the left M1 and patients who received sham stimulation and showed that pain was reduced and QoL was improved for up to 2–25 weeks after the treatment. In 2013, an RCT by Maetsu et al. (57) enrolled 54 patients with FM and showed that perceived chronic pain, somatosensory pain thresholds, sleep quality, and the ability to perform daily activities were improved in 28 patients who received low-intensity TMS (8 Hz) as compared with those in 26 patients who received sham stimulation. Most recently, in 2019, Altas et al. (60) recruited 30 patients with FM and applied 15 sessions of 10-Hz rTMS to the left M1 (10 patients), DLPFC (10 patients), and sham (10 patients). The results of this RCT showed that there were significant improvements in pain, QoL, and depression scores in all three groups. Notably, the decrease in VAS scores was significantly prominent in patients who received M1 rTMS stimulation, whereas improvement in physical function was significant in patients who received DLPFC rTMS stimulation. A similar RCT was performed in the same year by Cheng et al. (61) that included 20 patients with FM who also had major depressive disorder (MDD). This study showed that pain was significantly improved in patients who received 10-Hz rTMS over the left DLPFC (nine patients) over 2 weeks as compared with that in patients who received sham stimulations (11 patients). Interestingly, this study also conducted a subgroup analysis by depression severity and showed that the pain scores of patients who had mild-to-moderate depression were significantly decreased after receiving active rTMS treatments as compared with the pain scores of patients who had severe depression.

Conversely, three RCTs (53, 55, 59) opposed the beneficial effects of rTMS in patients with FM. A 2009 RCT conducted by Carretero (53) found that 1-Hz rTMS stimulation at the right DLPFC (14 patients) had no superior effect on pain reduction as compared with sham stimulation (12 patients) in 28 patients with FM and MDD. Two additional RCTs conducted in 2011 and 2015 (55, 59) reported that there were no significant differences in pain reduction between patients who received rTMS and those who received sham stimulations. The treatment in these two RCTs consisted of 10–15 sessions of 10-Hz rTMS that targeted the left DLPFC. In 2019, Abd Elghany et al. (62) prospectively recruited 120 patients with FM and compared the effectiveness of 10-Hz rTMS over the left DLPFC with prolotherapy. The results showed that the VAS scores and tenderness points were decreased in patients who received prolotherapy (60 patients) as compared with those in patients who received rTMS (60 patients); however, rTMS had better results on depression.

Despite the negative results of these three RCTs, the positive pain-reducing effects of rTMS on FM that were reported by some studies and the fact that FM pain is frequently refractory suggest that rTMS may be a possible therapeutic option for controlling pain associated with FM. Additionally, rTMS treatment may have beneficial effects on depression in patients with FM.

#### Headache

Migraine is a chronic neurovascular headache disorder characterized by severe headache attacks that require abortive therapy and prophylaxis for recurrent attacks to improve QoL. Studies have reported that the mechanisms of migraine are likely related to neural and vascular causes, including cerebral cell hyperexcitability, sensitization of the trigeminovascular pathway, genetics, and environmental factors (3). Posttraumatic headache is defined as a headache that develops after a head injury, including mild traumatic brain injury related headache (MBTI-HA) (122). Considering that rTMS has the potential to increase the activity of cortical structures that are involved in pain control or decrease cortical excitability, some studies have attempted to verify whether rTMS is effective for the treatment and prophylaxis of migraine or posttraumatic headache.

Our review identified 18 studies, including eight RCTs (63, 66–68, 70, 74, 76, 79), seven prospective observational studies (64, 69, 71, 72, 75, 78, 80), and three case studies (65, 73, 77), that evaluated the ability of rTMS to reduce the associated symptoms of headache, including frequency, duration, and severity of headache. The RCTs conducted by Brighina et al. (63), Lipton et al. (66), and Misra et al. (68) showed that outcome migraine measures, including headache frequency, VAS score, headache index, and number of abortive medications, were significantly reduced in patients with migraine who received rTMS treatment (10–20 Hz high-frequency or single-pulse TMS) as compared with those in patients who received sham stimulations. However, these results were contradicted by an RCT conducted by Teepker et al. (67) in 2010 and an RCT conducted by Conforto et al. (70) in 2013. Teepker et al. recruited 27 patients with migraine and applied low-frequency rTMS (1 Hz) over the vertex (14 patients were assigned to rTMS, and 13 patients were assigned to sham stimulation). Conforto et al. enrolled 18 patients with migraine and applied 10-Hz rTMS over the left DLPFC (nine patients received rTMS and the other nine patients received sham stimulation). Both studies revealed that the results after rTMS treatments were not superior to those after sham stimulations. Additionally, both studies showed a powerful placebo response. Interestingly, Leung et al. conducted two RCTs in 2016 and 2018 (74, 79) that included 24 and 29 patients, respectively, who had persistent MTBI-HA. In 2016, 10-hertz rTMS sessions (2,000 pulses/session) were applied at the left M1 (12 patients). In 2018, 10-Hz rTMS session were applied at the left DLPFC (14 patients). Both studies compared differences in headache intensity between patients who received rTMS and those who received sham stimulations and reported that persistent headache intensity was reduced in patients who received rTMS as compared with that in patients who received sham stimulation at the 4-week assessment after treatment.

We also identified seven observational prospective studies that investigated the positive effects of rTMS in patients with migraine (64, 69, 71, 72, 75, 78, 80). For example, Misra et al. (69, 78) found that plasma β endorphin levels were lower in patients with migraine than those in patients without migraine and that 10-Hz rTMS increased β endorphin levels. β endorphin levels above 4 ng/ml were associated with improvements in headache frequency in 43 of 93 patients (81.8%), and the increase of β endorphin levels correlated with headache relief. In 2015, Rapinesi et al. reported (75) that pain intensity and frequency of attacks were significantly reduced in seven patients with chronic migraine who received add-on 10-Hz rTMS over the DLPFC as compared with those in seven other patients who received standard pharmacotherapy.

In summary, the outcomes of the reviewed studies suggest that rTMS may be a beneficial treatment option for patients with migraine and MBTI-HA. However, the evidence for the most effective target site for high-frequency rTMS stimulation appears to be insufficient. Thus, more studies should be conducted to compare the effects of rTMS on the DLPFC with those on the M1 and also confirm the clinical effects of rTMS in patients with migraine or MTBI-HA.

#### Orofacial Pain

Patients with chronic orofacial pain usually present with complex pain and abnormal sensation disorders in the orofacial region. NP is a subtype of orofacial pain and includes trigeminal neuralgia and burning mouth syndrome (BMS). Persistent idiopathic facial pain, previously classified as atypical facial pain, remains a poorly defined category of pain that could be also categorized as an NP disorder (123).

Our literature search found eight studies (36, 81–87) that evaluated the effects of rTMS in patients with orofacial pain. One RCT (36), one prospective observation study (87), and one case study (82) included patients with trigeminal neuralgia, one RCT (86) and one case study (85) included patients with BMS, and two RCTs (83, 84) and one case study (81) included patients with non-specified orofacial pain. The RCT conducted by Khedr et al. involved patients with post-stroke pain syndrome (36) and also included 24 patients with trigeminal neuralgia. Results from this study revealed that pain reductions were improved in 14 patients who received 10 sessions of 20-Hz rTMS on the hand area of M1 as compared with those in 10 patients who received sham stimulation, and this effect lasted up to 2 weeks after treatment. In 2019, Henssen et al. (87) prospectively recruited 12 patients with trigeminal neuralgia who received bilateral and unilateral rTMS sessions in a random order. According to VAS scores, pain relief was greater in patients who received a 10-Hz rTMS session that targeted the bilateral M1 than that in patients who received a 10-Hz rTMS session that targeted the unilateral M1.

In 2015, Umezaki et al. applied a 10-Hz rTMS over the left DLPFC to one patient who had BMS and found that a 10-day treatment effectively decreased pain intensity of the tongue according to the VAS. In 2018, the authors conducted an RCT (86) that included 20 patients with BMS and revealed that the BMS pain intensity decreased by 67% in 12 patients who received 10 session of 10-Hz rTMS over the left DLFPC as compared with that in eight patients who received sham stimulation. Additionally, this pain reduction was observed immediately after 1 week of rTMS treatment, whereas no pain reduction was observed after sham stimulation.

In 2013, Fricova et al. (83) conducted an RCT that included 23 patients who had pharmacotherapy-resistant chronic facial pain resulting from trigeminal neuralgia, atypical orofacial pain, post-herpetic neuralgia, and dental pain. Thirteen patients received five sessions of 20-Hz rTMS over the contralateral M1, whereas 10 patients received sham stimulation. According to the VAS, pain intensity was effectively decreased in patients who received 20-Hz stimulation as compared with that in patients who received sham stimulation, and this effect lasted for 14 days. A 2015 RCT by Lindholm et al. (84) also enrolled 16 patients with neuropathic orofacial pain (7 with trigeminal neuralgia, 4 with atypical facial pain, and 5 with mouth burning syndrome). This was a crossover study, and each patient completed three treatment sessions, including sensorimotor (S1/M1), right secondary somatosensory (S2), and sham stimulations in random order. The NRS scores were significantly decreased after S2 stimulation as compared with those after S1/M1 stimulation. This finding suggested that the right S2 cortex may be a new target for treating neuropathic orofacial pain.

As there are few studies on orofacial pain, it is difficult to conclude whether rTMS is useful for this condition. However, it should be considered when patients do not respond to conventional treatment measures. Further studies on the effects of rTMS in patients with orofacial pain should be performed.

#### Phantom Pain

Phantom limb pain (PLP) is a neuropathic syndrome described as pain felt in the patients' remaining perception of an amputated limb and is characterized by a stabbing, throbbing, burning, or cramping sensation (93). PLP can be severe, intractable, and debilitating and occurs in up to 80% of patients after limb amputation (91, 124). Various treatments have been studied for treating PLP, including botulinum neurotoxin A, opioids, N-methyl D-aspartate receptor antagonists, antidepressants, anticonvulsants, and local anesthetics, but evidence regarding the efficacy of these treatments remains unclear (125). Maladaptive plasticity, which is associated with reorganization of the primary sensorimotor cortex, has been implicated in PLP development. This led to the use of rTMS to block maladaptive plasticity in the sensorimotor cortex (89). Considering the high prevalence of PLP after amputation and its lack of treatment response to conventional therapeutic approaches, studies have investigated the effect of rTMS as an alternative intervention for PLP.

Our literature search revealed two RCTs (89, 93) and five case studies (88, 90–92, 94) that reported that the application of rTMS effectively reduced the pain associated with PLP. In 2011, Ahmed et al. (89) applied 20-Hz rTMS to amputated patients with chronic PLP over the hand area of the M1 for 5 consecutive days. As compared with 10 patients who received sham stimulation, 17 patients who received rTMS reported long-lasting pain relief for up to 2 months, and serum β endorphin levels increased significantly after rTMS. In 2016, Malavera et al. (93) conducted an RCT with 54 land mine victims and compared 10 sessions of 10-Hz rTMS over the M1 to sham stimulation (27 patients in each group). The VAS scores of patients in the rTMS group were significantly decreased as compared with those in the sham group (-5 for rTMS and−2 for sham) 15 days after the treatment, but the effect had dissipated by 30 days after treatment. In addition, 19 patients in the rTMS group (70.3%) reported significant pain reduction (> 30%) 15 days after treatment. Five case studies (88, 90–92, 94) also reported that rTMS reduced pain in patients with PLP after amputation. High-frequency rTMS stimulation (10–20 Hz, mainly to the DLPFC) and low-frequency stimulation (1 Hz, targets including the supplementary motor complex, primary somatosensory area, and primary sensory cortex) were performed, and significant the patients reported significant improvements in PLP.

Overall, previous studies have shown the positive therapeutic effects of rTMS on PLP, suggesting that rTMS may be a beneficial method for alleviating PLP. However, more RCTs should be conducted before rTMS can become a standard of care for alleviating PLP.

#### Low Back Pain

Low back pain (LBP) is pain in the lumbosacral region and comprises a major worldwide health. The lifetime prevalence of LBP is 60–70% (95), and it is considered chronic when it persists for more than 3 months. Chronic LBP may provoke extreme suffering and deteriorate patient QoL. Abnormal postural control of the trunk muscles may contribute to this condition, and the M1 is assumed to have a critical role in postural control modulation (96, 126).

Our search located three studies, including one RCT (96) and two case studies (95, 97) that focused on the use of rTMS to treat LBP. In 2016, Ambriz-Tututi et al. (96) conducted an RCT to investigate the effectiveness of rTMS in patients with chronic LBP. Forty-one patients who received 20-Hz rTMS stimulation over the M1 experienced nearly an 80% reduction in pain from baseline by the 3rd week of treatment, which was significantly lower than that in patients who received sham rTMS (12 patients) or physical therapy (26 patients). Two case studies (95, 97) also reported that rTMS treatments decreased pain in four patients with chronic LBP and depression.

As traditional pharmacotherapy (e.g., non-steroidal anti-inflammatory drugs, muscle relaxants, or antidepressants) and other interventions (e.g., physical therapy or TENS) may be ineffective in some cases of LBP, rTMS can be applied for additional benefits for pain reduction and restoration of the normal postural control networks in the M1. Despite the previous studies showing that rTMS is beneficial for this condition, more definitive evidence is needed.

#### Myofascial Pain Syndrome

Myofascial pain syndrome (MPS) is one the most common causes of musculoskeletal pain. It is characterized by the presence of one or more hypersensitive sites, which are also known as trigger points. The trigger points are surrounded by an area where the muscle appears as a tight and rigid stricture, and this is referred to as a “taut band” (127). Numerous therapeutic approaches are used to treat MPS, including education, acupuncture, massage, ultrasonography, electrotherapy, dry needling, drugs, and physiotherapy rehabilitative treatments (124). As chronic pain caused by MPS is induced by central and peripheral sensitization, a neuromodulatory technique, including rTMS, may aid in modulating this sensitization process by reverting the associated defective inhibitory systems (99).

Two RCTs (98, 99) have investigated the effect of rTMS on patients with MPS. In 2014, Dall'Agnol et al. (98) applied 10 sessions of rTMS treatment to 24 patients who were diagnosed with MPS in an upper body segment for at least 3 months (12 patient received real rTMS, and the remaining 12 received sham rTMS). The results showed that daily pain scores were reduced by 30% and analgesic use was reduced by 45% in patients who received 10-Hz rTMS at the left M1 as compared with those in patients who received sham rTMS. Interestingly, in 2016, an RCT conducted by Medeiros et al. (99) attempted to determine whether 10-Hz rTMS and deep intramuscular stimulation therapy (DIMST) would be effective in patients with MPS. In this study, 44 patients were divided into four groups (11 patients per group) and allocated to receive rTMS only, DIMST only, both rTMS and DIMST, or sham. Patients received 10 sessions of each for 20 min and pain relief was assessed by the VAS immediately after the intervention. The results showed that the VAS scores in all three active groups were lower than those in the sham group, but no synergistic effect was observed in the rTMS and DIMST group.

Collectively, the outcomes of these two RCTs revealed that rTMS can also be considered as a beneficial treatment option for patients with MPS. However, the results that indicated that significant pain reduction was achieved after rTMS have to be reproduced in other well-designed RCTs before rTMS can become a tool of care for MPS.

#### Pelvic Pain

Bladder pain syndrome (BPS), also known as “interstitial cystitis,” is a relatively common disease that is characterized by suprapubic pain related to bladder filling; pain throughout the pelvis; and pain in extragenital locations, including the lower abdomen and back. BPS is also frequently accompanied by urinary symptoms, including urgency, frequency, and nocturia (101, 128). BPS is the cause of pain in more than 30% of women with chronic pelvic pain (CPP) (100). The mechanism of bladder pain and urinary problems is hypothesized to be associated with the misperceptions and improper integration of sensory information provided by bladder filling and central sensitization (101).

Our search identified one RCT (100), one prospective observational study (102), and one case report (101) that investigated the efficacy of rTMS for treating pelvic pain. In a crossover RCT conducted by Cervigni et al. (100), 13 patients were randomized into two groups. The first group (seven patients) received 20-Hz rTMS sessions over the M1 in the area corresponding to the pelvic region for 20 min for 2 weeks, and this was followed by sham treatment. The second group received the same treatments in an inverted order. According to the VAS scores and associated urinary symptoms measured by an overactive bladder questionnaire, CPP was significantly improved after the real rTMS stimulation phase as compared with CPP after the sham stimulation phase. The authors assumed that rTMS probably modulated brain plasticity through a process of functional reorganization of the neuronal connections at the cortex level and modified the excitability of sub-cortical areas. In the same year, Nizard et al. (101) reported successful application of 1-Hz rTMS delivered on the DLPFC region of both hemispheres in one patient, which resulted in complete resolution of suprapubic pain and a dramatic decrease in micturition frequency. Most recently, in 2019, Pinot-Monange et al. (102) prospectively enrolled 12 patients with refractory CPP caused by endometriosis. After five 10-Hz rTMS sessions targeting the left M1 over the hand representation, nine patients reported pain improvements as measured by the VAS score, and these improvements lasted up to 28 days.

Despite the favorable treatment outcomes in these previous studies, the usefulness of rTMS in pelvic pain should be investigated further and studies should include larger subject populations to clarify its efficacy.

#### Complex Regional Pain Syndrome

Complex regional pain syndrome (CRPS) is a chronic pain condition defined as “continuing pain, which is disproportionate to any inciting event,” along with other signs and symptoms, including sensory, vasomotor, sudomotor/edema, and/or motor/trophic changes (129). It is subdivided into two types: CRP type I (no peripheral nerve lesions) or type II (definable nerve lesion). Postulating that pain perception can be modulated by rTMS, several studies have investigated the analgesic efficacy of rTMS in patients with CRPS.

Our search revealed one RCT (104) and two prospective observational studies (103, 105) that used rTMS in patients with CRPS. A 2010 RCT conducted by Picarelli et al. (104) included patients with CRPS type 1 of the upper limb who were treated medically. Ten daily sessions of 10-Hz rTMS to the M1 or sham stimulation (11 patients in each group) were added to the pharmacotherapy. The results showed that the VAS scores in the TMS group were lower than those in the sham group. The highest reduction occurred at the 10th session. Two prospective observational studies conducted in 2004 (103) and 2018 (105) also showed that high-frequency rTMS offered short-term pain relief for CRPS. This effect was shown 30 seconds after stimulation and was maximized 15 min later (103). Additionally, the reduction in pain was maintained beyond 1 week post-treatment (105). Thus, rTMS may be a useful tool to alleviate pain in patients with CRPS.

However, more definitive evidence is needed to clarify the therapeutic effect of rTMS for managing pain induced by CRPS.

#### Other Disorders

In addition to the above disorders and conditions, rTMS has been used to treat other painful conditions, including postherpetic neuralgia (PHN), brachial plexus injury, postsurgical pain, chronic visceral pain, somatoform pain disorders (SPD), knee osteoarthritis (OA), and hemiplegic shoulder.

PHN is NP that occurs as a complication of herpes zoster. As treating PHN with conventional analgesics is challenging, a 2015 RCT by Ma et al. (112) enrolled 40 patients with PHN and randomly assigned them to receive 10 sessions of 10-Hz rTMS to the precentral gyrus or sham stimulation (20 patients in each group). The results showed that the VAS scores were reduced in patients who received rTMS as compared with those in patients who received sham stimulation, and this lasted for up to 3 months. The mean VAS reduction was 16.89% after real rTMS, and no significant changes were seen after sham stimulation.

Brachial plexus avulsion is often followed by chronic pain in the deafferented area and is characterized by constant unbearable pain. Our literature search showed that three case studies (14, 109, 111), each reporting a case of one patient, investigated the effectiveness of rTMS for patients with chronic pain due to a brachial plexus injury. Patients with chronic intractable deafferentation pain (111), allodynia (109), and drug-resistant NP (14) caused by brachial plexus injury received high-frequency rTMS sessions (10–20 Hz) over the M1 and reported that they experienced pain relief after the treatment.

Postsurgical pain is associated with high levels of opioid medication use, which is associated with serious side effects (106). Three RCTs conducted by Borckardt et al. (106, 107, 110) investigated if rTMS significantly reduced acute postsurgical pain and consequent opioid use. These studies included patients who had undergone gastric bypass surgery. In the 2006 study (106), 20 patients were allocated to receive a single session of either 10-Hz rTMS (total 4,000 pulses/session) or sham rTMS (10 patients in each group). The results showed that morphine use was reduced by 40% in patients who received real rTMS as compared with that in patients who received sham rTMS, and this effect occurred within the first 24 h after stimulation. A replication study was performed in 2008 (107); 12 patients were assigned to the real rTMS group and eight patients were assigned to the sham group with the same rTMS protocol. The results were the same as those from the study conducted in 2006. However, a large-scale RCT conducted in 2014 (110) that included 108 patients undergoing laparoscopic gastric bypass surgery contradicted these results. Patients were randomly assigned to receive two sessions of real rTMS (28 patients), two sessions of sham (28 patients), one real then one sham rTMS (27 patients), or one sham then one real rTMS (25 patients) treatment. The results revealed that there were no differences in total patient-controlled analgesia usage of morphine between patients who received two sessions of 10-Hz rTMS or sham treatment.

Chronic visceral pain is extremely refractory and debilitating and often resistant to pharmacological and surgical treatments (130). Altered afferent visceral sensory input and neuroplastic changes in the spinal cord and brain may be responsible for sustained chronic visceral inflammation. In an attempt to control visceral inflammation by modifying activity in the CNS, Fregni et al. conducted an RCT in 2011 that included 17 patients with visceral pain due to chronic pancreatitis (108). Ten sessions of 1-Hz rTMS over the right S2 (total 1,600 pulses/session) were applied to nine patients, whereas eight patients received sham stimulation. Real rTMS induced a 27.2% decrease in pain levels as measured by the VAS, whereas sham rTMS only induced a 1.1% increase in pain levels. This effect was sustained for at least 3 weeks after real rTMS. These results suggested that rTMS may be useful in refractory cases of chronic visceral pain.

Somatoform disorders, including somatoform pain disorders (SPD) are generally recalcitrant and associated with poor QoL (131). Considering the challenges in managing SPD, a case study was reported in 2018 by Singh et al. (113). In this study, 18 sessions of 10-Hz rTMS (total 1,200 pulses/session) were applied to five patients with SPD. After rTMS, patients reported reductions in VAS scores and that their pain had greatly decreased. All of the patients, except for one, reported improvement of at least 62.5%. This effect was sustained at 2 weeks after the last session of rTMS. Thus, rTMS was suggested to be useful in alleviating pain associated with SPD.

Although knee OA is related to inflammation of the joint and anti-inflammatory treatments are most commonly used in the clinical setting, in some cases of knee OA, pain extends beyond the joint and acquires neuropathic features. The repetition of painful episodes may be due to the sensitization to pain in the CNS. Since rTMS has been proven to be useful in chronic pain conditions associated with CS, Nguyen et al. (114) reported that the pain of one patient who was treated with 10 monthly sessions of 10-Hz rTMS over the right motor cortex was reduced by 67%. This finding suggested that rTMS may be an alternative therapeutic option for treating NP associated with CS in patients with chronic OA.

Hemiplegic shoulder pain is one of the most common causes of pain after stroke and develops in 87% of patients at 4 months after stroke. In 2018, Choi et al. (11) conducted an RCT that included 24 patients with hemiplegic shoulder pain. Twelve patients were allocated to receive 10-Hz rTMS over the contralateral motor cortex, and the other twelve received sham stimulation. The NRS scores of patients who received rTMS were significant decreased on day 1 (30.1%) as compared with the NRS scores of patients who received sham stimulation, and this effect lasted for up to 4 weeks (25.3%) after the rTMS sessions.

## Conclusion

This review showed that rTMS may be an alternative treatment method for patients with chronic pain conditions. rTMS is not associated with serious complications and appears to be beneficial for treating NP of various origins, including central pain and pain from peripheral nerve disorders, FM, and migraine. Additionally, rTMS may be a valid treatment for patients with orofacial pain, including trigeminal neuralgia, PLP, LBP, MPS, pelvic pain, and CRPS. rTMS may also be an alternative treatment option for pain relief in some cases of PHN, brachial plexus injury, postsurgical pain, chronic visceral pain, SPD, knee OA, and hemiplegic shoulder.

Overall, rTMS appears to be effective for short-term pain relief, but the long-term effects of rTMS on pain relief (>3 months) should be investigated further. To clarify the usefulness of rTMS in managing pain induced by the various conditions mentioned above, numerous well-designed RCTs are needed to validate the positive effects of rTMS on pain relief. Further, various factors related to rTMS, including the stimulation frequency, stimulation site, and treatment duration, can affect the results of rTMS. Accordingly, further studies that investigate the most appropriate rTMS mode for each type of pain should be conducted. Our review provides insights on the degree of evidence according to pain from each disorder, which can help clinicians decide when rTMS should be used to treat various types of pain. Our study is limited in that we were not able to directly compare the effects of rTMS between each pain disorder because different outcome measurements were used in different studies.

## Author Contributions

SY: study concept and design, manuscript development, and writing. MC: writing and critical revision of manuscript for intellectual content.

### Conflict of Interest

The authors declare that the research was conducted in the absence of any commercial or financial relationships that could be construed as a potential conflict of interest.
